# Factors affecting motivation and retention of primary health care workers in three disparate regions in Kenya

**DOI:** 10.1186/1478-4491-12-33

**Published:** 2014-06-06

**Authors:** David Ojakaa, Susan Olango, Jordan Jarvis

**Affiliations:** 1 AMREF Kenya, Wilson Airport, Langata Road, PO Box 30125, Nairobi, Kenya; 2 Formerly with AMREF Kenya, Wilson Airport, Langata Road, PO Box 30125, Nairobi, Kenya; 3 Formerly with AMREF Canada, 489 College Street West, Suite 403, ONM6G 1A5 Toronto, Canada

**Keywords:** Motivation, Retention, Kenya, Health care workers

## Abstract

**Background:**

The World Health Organization (WHO) and the Government of Kenya alike identify a well-performing health workforce as key to attaining better health. Nevertheless, the motivation and retention of health care workers (HCWs) persist as challenges. This study investigated factors influencing motivation and retention of HCWs at primary health care facilities in three different settings in Kenya - the remote area of Turkana, the relatively accessible region of Machakos, and the disadvantaged informal urban settlement of Kibera in Nairobi.

**Methods:**

A cross-sectional cluster sample design was used to select 59 health facilities that yielded interviews with 404 health care workers, grouped into 10 different types of service providers. Data were collected in November 2011 using structured questionnaires and a Focus Group Discussion guide. Findings were analyzed using bivariate and multivariate methods of the associations and determinants of health worker motivation and retention.

**Results:**

The levels of education and gender factors were lowest in Turkana with female HCWs representing only 30% of the workers against a national average of 53%. A smaller proportion of HCWs in Turkana feel that they have adequate training for their jobs. Overall, 13% of the HCWs indicated that they had changed their job in the last 12 months and 20% indicated that they could leave their current job within the next two years. In terms of work environment, inadequate access to electricity, equipment, transport, housing, and the physical state of the health facility were cited as most critical, particularly in Turkana. The working environment is rated as better in private facilities. Adequate training, job security, salary, supervisor support, and manageable workload were identified as critical satisfaction factors. Family health care, salary, and terminal benefits were rated as important compensatory factors.

**Conclusions:**

There are distinct motivational and retention factors that affect HCWs in the three regions. Findings and policy implications from this study point to a set of recommendations to be implemented at national and county levels. These include gender mainstreaming, development of appropriate retention schemes, competitive compensation packages, strategies for career growth, establishment of a model HRH community, and the conduct of a discrete choice experiment.

## Background

Kenya currently faces significant challenges in overcoming health care worker (HCW) shortages and low HCW retention, as well as difficulty attaining equitable distribution of human resources for health (HRH) - particularly in hard-to-reach areas. A 2008 report indicates that the Ministry of Health had an overall vacancy level of 29% [1,2]. There are 1.5 HCWs per 1,000 population in Kenya, which falls below the figure of 2.3 per 1,000 population reported in analyses by the World Health Organization (WHO) on the minimum staffing threshold to achieve minimum coverage [1,2]. Vacancy levels based on WHO suggestions were highest in Northern Kenya, with 85% and 93% health worker vacancy levels in Turkana and Mandera -counties, respectively [3].

Health worker motivation (defined as the extent an individual is willing to exert and maintain effort towards the achievement of an organization’s goals) has frequently been cited as a critical barrier to effective health service delivery and contributor to the HCW shortage [4-6]. In this regard, several themes characterize motivation and these include financial aspects, career development, continuing education, health facility infrastructure, availability of resources, relationships with the management of the health facility, and personal recognition. Further, there is an urgent need to ascertain and employ successful retention strategies that are suitable for different regions with diverse needs [7], where retention strategies are commonly understood to mean incentive mechanisms provided to health care providers already working in rural (and remote) areas to continue working in these regions.

Motivation is closely tied to job satisfaction (a positive feeling which results from evaluating one’s job) and neither of these is directly observable, but both are critical to the retention and performance of health workers [8,9]. A study on health worker motivation in Kenyan district hospitals demonstrated that altruistic motives are important in these settings, but that their organizational commitment (in terms of decisions on performance on the job depending on whether the senior management/the organization appreciated the particular staff or not) and motivation was threatened by the many challenges that service providers face in public sector health care provision [10]. This further highlights the need to evaluate differences in the motivation and retention of HCWs between private and public health facilities, since challenges faced in these facilities often differ [11,12]. In this case, private facilities encompass faith-based organization (FBO), non-governmental organization (NGO), and private for profit health facilities that are owned by individuals or corporates. This said, one important unifying framework for analyzing factors in the motivation and retention of health workers is Herzberg’s hygiene and motivation theory on job satisfaction [13]. At the operational level, the HRH action framework (which includes the human resources management systems) helps to address issues related to staff shortages, uneven staff distribution, skill and competency gaps, low retention, and poor motivation [14].

Health workers are susceptible to ‘push’ factors, such as pay and working conditions, and ‘pull’ factors, such as job satisfaction and economic prospects [15,16]. Ensuring that staff receive adequate pay for their work is key to retention. However, salary is not the only important dimension [5,15]. In many contexts, the low numbers of trained health staff in remote areas is due to the lack of supporting infrastructure and opportunities for staff and their families [17,18].

In fragile environments, these factors include poor living conditions, the lack of safety and security in the workplace, and the absence of continuous professional development [1,4]. Observations from Malawi also show that continuous education and progressive career growth are not sufficient in the retention of health workers, and that strong human resource management (HRM) practices lead to enhanced HCW motivation and performance [19]. These HRM practices include performance appraisal, presence of job descriptions, adequate supervision, and feedback on performance.

Several reasons explain attrition of health workers in Kenya. These include retirement, resignation, and death [15]. A number of critical factors contribute to the motivation and retention of staff, yet these are not currently well understood in the Kenyan context.

Moreover, Kenya is a diverse country both culturally and geographically, with different working conditions in distinct regions. Without a clear understanding of the various factors that affect health care worker motivation and retention in different contexts [20], it is likely that communities will continue to face challenges in receiving accessible and high quality primary health care. This study examined factors that lead to motivation and retention of health workers at the primary health care level in three disparate regions of Kenya: Turkana, Machakos and Kibera, Nairobi.

These sites are significantly varied and offer a wide scope for implementation, learning and innovation. The remote and arid Turkana is a county located in the north of Kenya and a hardship area, whereby hardship area is defined to mean a location where living is relatively harder, for example due to being a very dry region and hence inadequate water, or a region of conflict implying restricted movement. In Turkana County, the community derives its livelihood from nomadic pastoralism. Machakos County is composed largely of agro-pastoralists and falls within the relatively accessible rural Eastern region. Lastly, Kibera is an informal urban settlement, comprising the most socioeconomically disadvantaged groups in Nairobi. Given the implementation of the new constitution in which the new County Governments now control the implementation and management of most development activities including health service delivery, the findings from this study are applicable first and foremost in the respective counties of Turkana, Machakos, and the slum/informal settlements in Nairobi. Additionally, the results should be applicable to the surrounding districts with similar socioeconomic and environmental conditions to each of the three counties. For Turkana, these would include parts of West Pokot and Samburu; for Machakos, these would incorporate the other Kambaland counties of Kitui and Makueni; for the informal settlements, the findings could be considered for other slum areas in other bigger cities of Kenya namely Mombasa, Nakuru, and to some extent Kisumu cities.

Currently, Kenya’s health system for the public sector is organized around six levels which are the community (level 1), the dispensary (level 2), the health centre (level 3), the sub-county and county hospitals (level 4), the regional hospitals (level 5), and the national referral and teaching hospitals (level 6). A large part of health service delivery is the responsibility of the 47 county Governments, while the national level Ministry of Health is expected to focus on policy, guidelines, and training. A Human Resource Mapping and Verification Study conducted in 2004 indicated that there were 35,643 health workers in the public sector with more female (52.7%) than male (47.3%) workers. Of these, enrolled nurses as a group are the largest in number contributing 48.3% of the entire health workforce.

The distribution of healthcare providers in Kenya has been skewed against many rural areas, with many doctors found in the urban areas and fewer in rural facilities. The Kenya National Human Resources for Health Strategic Plan 2009 to 2012 identified five critical outcomes, one of which is improved retention of health workers at all levels [1]. Two objectives Kenya has set to attain in order to reach this outcome are: making health sector jobs more attractive in order to improve staffing levels and reduce attrition, and making hard-to-reach areas more attractive to health workers [1]. Currently, Kenya is finalizing its second HRH strategic plan and to retain staff in rural and hard to reach areas, two strategies have been outlined. These are making these areas more attractive to health workers, and improving the compensation package for these workers. Appropriate policies to retain staff in the health sector need to be tailored for different cadres, contexts, level and type of health facility [20]. Our study sought to bridge the knowledge gap that currently exists on the retention and attraction of health workers in Kenya, particularly in underserviced communities, in order to inform the tentative 2013 to 2018 national HRH strategy and programme development at the national and county level.

## Methods

### Sample design

Three methods of data collection were used in the study: a questionnaire that was self-administered by the service providers and in-charges of facilities, face-to-face interviews with key informants, and Focus Group Discussions (FGDs) with support staff and service providers. This represents an overall mixed-methods design and was necessitated by the need to obtain richer information around the close-ended questionnaires posed in the quantitative structured interview. The design of the quantitative part of the study was cross-sectional, with a cluster sample design [21,22] of facilities sampled from the national facility frame, focusing on the regions of Turkana, Machakos, and Kibera in Nairobi. The study population consisted of all health workers present at survey time in the selected health facilities across the regions of the study. Interviews were conducted in each of the health facilities visited - all the staff present at the facility at the time of the interview participated in their respective interviews, after the District Health Management Team (DHMT) and Field Research Coordinators informed the in-charge of the health facility of the visit for interviews. Although all staff present in the facility were interviewed, the survey particularly targeted the technical service providers rather than support staff *per se*. A practice observed in several of the health facilities (particularly dispensaries) is that support staff take some responsibility for the facility when the service providers (such as the in-charge) are away on leave. This may partly explain the significant number of support staff in the samples, particularly for Turkana. When administering the questionnaires, interviewers were careful to ensure that each respondent was provided with free space to fill out the questionnaire independently. Health workers comprised technical and non-technical staff, which includes medical officers (physicians), nurses, clinical officers, laboratory officers and clerks, as well as support staff.

All operational level 2 and 3 health facilities (dispensaries and health centres) in the selected divisions were of interest. This consisted of a total of 208 health facilities across the three regions. A cluster sample size of 59 health facilities was statistically calculated and selected. The selection process ensured that the selected sites were well distributed geographically. The number of health facilities selected per region was 26 in Turkana, 17 in Kibera, and 16 in Machakos. This resulted in representation of both public and private health facilities. In each of the three regions, two FGDs were conducted with support staff and service providers, respectively. The qualitative data (FGDs and key informant interviews) were tape-recorded, transcribed, and analyzed manually to reveal themes and typologies. Data collection took place in November 2011. The questionnaire used in this study was adopted from the worker satisfaction measurement tool developed by the Capacity Project, previously used in the Uganda Health Workforce Study [23]. Satisfaction factors were evaluated by responses made on a five-point scale: ‘strongly agree’, ‘agree’, ‘neutral’, ‘disagree’ or ‘strongly disagree’. The same scale was used with statements about working conditions and remuneration comparing the three regions, and compensation factors by type of health facility. Responses on the importance of compensation factors by region were evaluated based on a three-point scale: ‘not important’, ‘somewhat important’ or ‘very important’.

### Respondents

The cluster design meant that all staff found in an elected health facility were interviewed. A total of 404 respondents participated in the interview (Table 1). Nurses (registered and enrolled) formed the majority of health care workers at 28.7%. Machakos had the highest proportion of both nurses and clinical officers, which accounted for 34.8% and 11.9% of health care workers, respectively. In Turkana 57% of workers were support staff, compared to 30% in Machakos and 32% in Nairobi. A higher proportion of health workers (51%) were from private versus public health facilities.

**Table 1:** Percentage distribution of types of service providers by region

**Type of service provider**	**Nairobi (n = 171) %**	**Machakos (n = 135) %**	**Turkana (n = 98) %**	**Total (n = 404)**
Registered nurse	19.9	32.6	15.3	23.0
Enrolled nurse	8.8	2.2	5.1	5.7
Lab technician	10.5	7.4	4.1	7.9
Clinical officer	8.2	11.9	5.1	8.7
Nutritionist	2.3	1.5	4.1	2.5
Medical officer	2.3	0.7	0.0	1.2
Counsellor	7.0	3.7	3.1	5.0
Pharmacist	4.7	2.2	2.0	3.2
CHEW	4.1	7.4	4.1	5.2
Support staff	32.2	30.4	57.1	37.6
Total	100.0	100.0	100.0	100.0

CHEW, community health extension worker.

### Data collection

Both quantitative and qualitative data collection instruments were designed and developed by AMREF staff. To ensure quality and validity of these tools, a pre-test was conducted in health facilities outside of the study sites. Following a structured questionnaire, a trained research assistant conducted face-to-face interviews with every enrolled participant to measure factors affecting health worker motivation and retention. Qualitative information was also collected through FGDs for each of the three regions.

During the interviews, critical variables for the study were recorded in an interviewer- administered structured questionnaire. These variables include individual factors (demographic, training background, and socioeconomic); aspects related to the context, neighborhood or environment; factors related to motivation. Outcome variables of interest (job satisfaction, whether the respondent had changed jobs in the past 12 months, intent to leave the job in future) were also captured.

### Data analysis

The study focused on the effects of individual characteristics, working conditions, context, and incentives on the motivation and retention of health workers. Dependent (duration of stay in current job and job satisfaction) and independent variables (such as background characteristics, financial incentives, and work environment factors) were chosen to determine factors affecting job satisfaction and attrition.

Three methods of data analysis were applied in this study. Qualitative data were managed and analyzed in descriptive form. Responses on the five-point scale were analyzed by calculating percentages for each category in the scale rather than estimation of the median scores. From the results generated, it is clear that there are specific distinct categories which stand out, which counters the argument that the five-point scale usually tends to register the middle scores. Quantitative data were processed and analyzed using SPSS (SPSS Inc., Chicago, IL, USA) and STATA (Cambridge, MA, USA).

### Ethical considerations

This study was approved by the African Medical and Research Foundation Ethics and Scientific Review Committee (Registration number NCST/NBC/AC/0912). All respondents were informed about the purpose of the survey and gave informed consent prior to study participation.

## Results

### Background characteristics of respondents

A total of 404 participants enrolled in the study across three regions: Nairobi (n = 171), Machakos (n = 135), and Turkana (n = 98) (Table 2). In Nairobi and Machakos, approximately twothirds of participants were female, while the reverse was observed in Turkana. Overall, the sample included 234 (57.9%) females and 170 (42.1%) males. About 67% of all the respondents were aged 35 years or less, with the proportion in Turkana being highest at 72%. Further analysis of the age distribution by five age categories provided the following results: less than 25 years of age, 8%; 25 to 29, 31%; 30 to 34, 22%; 35 to 39, 15%; 40 to 44, 9%; 45 to 49, 6%; 50 to 54, 6%; those approaching retirement (55+), 3%. The median duration in years since qualification was eight years for all respondents, being highest at nine years for Nairobi, seven for Machakos and five years in Turkana. The proportion of respondents that were married was 65.8% overall, with Turkana having the highest number of married participants at 73.5%. The number of children respondents had was also higher in Turkana than in the other regions (three children versus two at other sites). Completion of post-secondary education was at 80% of all participants, with the lowest level of post-secondary education recorded in Turkana (56.6%), compared to Nairobi (90.6%) and Machakos (90.3%). Most of the workers without post-secondary education were support staff. However, some support staff- mainly in Nairobi-have degree-level education.

**Table 2:** Background characteristics of respondents

**Characteristic**	**Category**	**Nairobi (n = 171) %**	**Machakos (n = 135) %**	**Turkana (n = 98) %**	**Total (n = 404) %**
Sex	Male	38.0	27.4	69.4	42.1
	Female	62.0	72.6%	30.6	57.9
Age	≤35 years	62.7	68.1	72.4	66.9
	>35 years	37.3	31.9	27.6	33.1
Education	Post secondaryeducation	90.6	90.3	56.6	80.2
Marital status	Married	67.3	58.2	73.5	65.8
	Unmarried	32.7	41.8	26.5	34.2
	Farming	12.1	27.5	1.0	14.5
Alternative sources of income	Business	10.9	12.2	20.6	13.7
	Consultation^1^	1.8	0.0	0	0.8
	Part-time job^2^	0.0	3.1	13.4	4.3
	Other^3^	75.2	57.3	64.9	66.7

^1^Consultation here refers to provision of health services, advice, or consultancy for a fee and for a specified short duration of time.

^2^Part-time job means working some of the time during the day, week, or month; usually an agreement or contract is signed.

^3^The ‘other’ category comprises a large percentage, possibly because of the diverse activities that the many support staff in this study are involved in.

Across all 3 settings, 52% of participants reported that their salary was the major source of income. Other sources of income for health care workers (HCWs) included farming (14.5%), business (13.7%), consultation (0.8%) and part-time jobs (4.3%). While business was the preferred source of alternative income in Turkana (21%), farming was more common in Machakos (27%) and Nairobi (12%). Only Nairobi had consultancy as an alternative source of income (1.8%).

### Comparisons by region

#### Training qualifications of professional health care workers

Within the category of professional health workers, which includes nurses, laboratory technicians, clinical officers, nutritionists, medical officers, counsellors, pharmacists and community health extension workers (CHEWs), 64.6% had at least diploma-level training (Table 3). Significantly more HCWs had diploma-level or higher education in Machakos (55.8%) and Nairobi (52%), than in Turkana (48.2%) (*P*< 0.0001). In terms of upgrading training courses, a higher proportion of respondents in greater Machakos (67.8%) had attended such training, in comparison to 52.3% in Nairobi and 40% in Turkana. More than half of the respondents who had attended an upgrading course did so within the past 12 months.

**Table 3:** Training characteristics of professional health care workers

**Characteristic**	**Category**	**Nairobi (n = 100) %**	**Machakos (n = 68) %**	**Turkana (n = 27) %**	**Total (n = 195) %**
Highest level of training	<Diploma	28	41.2	48.1	35.4
	Diploma	52	55.8	48.2	52.8
	>Diploma	20	3	3.7	11.8
Attended upgrading course	Yes	(n = 111)	(n = 87)	(n = 40)	(n = 238)
		52.3	67.8	40	55.9
	No	47.7	32.2	60	44.1

Note: Data from some participants were excluded due to incomplete or inaccurate completion of the questionnaire.

#### Job satisfaction

For all the three regions, 44.3% of all respondents agreed or strongly agreed that considering everything, they were satisfied with their job [see Additional file 1]. Although this was highest in Nairobi (48.4%), the chi-square test did not however show any significant difference between the respective percentages for the three regions. About 66% of HCWs felt that their supervisors provide support and encouragement, but the percentage differences were not significant between the three. When asked whether participants felt that they had been given sufficient training to perform their expected duties, 46.5% of interviewees stated that they agree or strongly agree. The proportion of participants who agreed or strongly agreed that they had been provided the necessary training for their position was highest in Nairobi at 60.2%, less in Machakos (43.7%) and least in Turkana (27.3%) (*P*< 0.0001). Qualitative interviews from FDGs further revealed that staff shortages, transportation, inadequate supportive supervision from the county or district level, and insufficient essential supplies contributed to dissatisfaction of HCWs.

#### Work environment

Comparing the three sites, 26.3% of respondents from Turkana, 24.3% from Machakos and 14.8% from Nairobi said the workload was not manageable [see Additional file 2]. Overall, 43.2% of respondents indicated that they did not have necessary equipment available to them, a proportion that was higher in Turkana (63.3%) than in Machakos (23.2%) and Nairobi (52.7%) (<0.0001). About 74% of respondents indicated that they are allowed to take annual leave, but this proportion was lower in Machakos (62.8%) than in Turkana (70.1%) or in Nairobi (83.9%). In Machakos, 45.2% of respondents reported feeling a lack of job security, compared to 27.4% of those in Turkana and 27.6% in Nairobi. A higher proportion of respondents in Machakos indicated that their children did not have access to good schooling, compared to those in Turkana and Nairobi. Access to safe water at the work place was a bigger problem in Machakos (45.1%) and Turkana (32.3%) than in Nairobi (17.6%). During FDGs, several factors were highlighted as non-conducive to a productive work environment: lack of suitable housing, insufficient payment of support staff, and deplorable physical state of the health facility.

#### Distribution of remuneration factors

In both Turkana and Nairobi, approximately 30% of HCWs felt that their salary package was fair, whereas only 5.9% of HCWs in Machakos agreed to receive a fair salary (Table 4). The proportion of respondents who felt that opportunities for promotion were available was highest in Nairobi at 38.7%, followed by Turkana at 27.3%, and Machakos at 26.3%.

**Table 4:** Health care worker responses to remuneration factors in Nairobi, Machakos and Turkana

		**Nairobi (n = 168) %**	**Machakos (n = 135) %**	**Turkana (n = 98) %**	**Total (n = 401) %**
My salary package is fair	Strongly disagree	19.0	45.9	23.2	29.1
	Disagree	26.8	31.1	29.3	28.9
	Neutral	24.4	17.0	17.2	20.1
	Agree	21.4	4.4	26.3	16.9
	Strongly agree	8.3	1.5	4.0	5.0
There are sufficient opportunities for promotion	Strongly disagree	13.1	17.8	20.2	16.4
	Disagree	16.1	23.0	32.3	22.4
	Neutral	32.1	23.0	20.2	26.1
	Agree	27.4	25.9	18.2	24.6
	Strongly agree	11.3	10.4	9.1	10.4

#### Compensation factors

**Figure 1:**
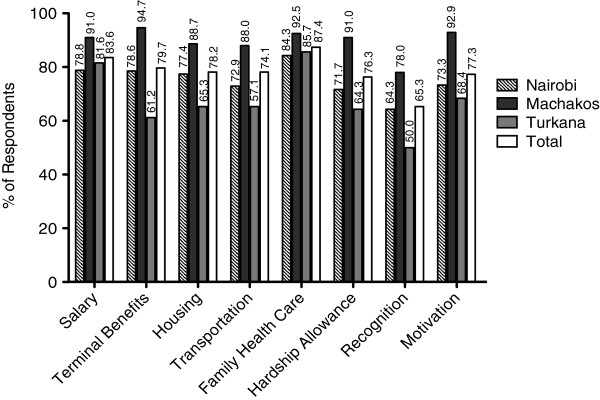
**Health care workers’ rating of compensation factors in three regions.** Percent of respondents in Nairobi (n = 168), Machakos (n = 133) and Turkana (n = 98) who rated select compensation factors as very important. Please note: (1) ‘recognition’ here refers to ‘recognition and awards scheme’; (2) ‘motivation’ means ‘measure of motivation instituted by the organization’; (3) ‘terminal benefits’ refer to payments made to the worker as they finally leave the organization, for example on retirement. Terminal benefits include payment of pension; (4) Health care for family in the strictest sense refers to inclusion of dependants in the medical cover and insurance for family members.

Among compensation factors considered very important to HCWs, family health care was considered most important across all regions (87.4% total rated it as very important), followed by salary (83.6%) and terminal benefits (79.3%) (Figure 1). Out of the three regions, respondents in Machakos consistently reported all compensation factors under consideration with highest importance, whereas Turkana respondents least reported these factors as very important. During qualitative interviews, HCWs also reported allowances (hardship, marriage, over-time), as well as rest and recuperation time as being important.

#### Retention of health care workers

**Figure 2:**
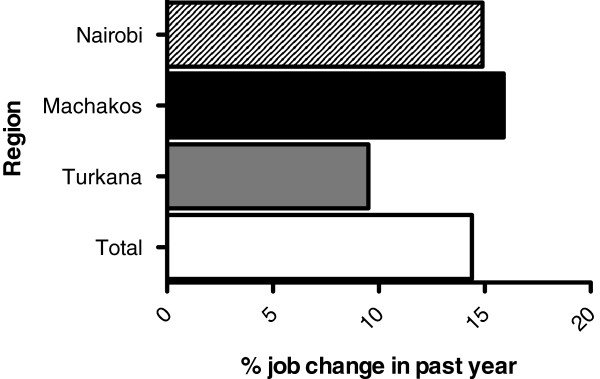
**Proportion of health workers who reported changing their job in the past year by region.** Job change was reported by 14.91% of respondents in Nairobi (n = 167), 15.96% in Machakos (n = 135), 9.52% in Turkana (n = 98) and by 14.4% of total respondents.

Figure 2 shows that the number of health care workers who have changed jobs in the past year was comparable between Machakos (15.6%) and Nairobi (15%), but lower in Turkana (6.1%). A higher proportion of those who changed jobs were professional health workers (85.7%), as opposed to support staff (14.3%).

The duration health workers would stay in their current employment was measured against several background characteristics, work environment, and remuneration and compensation predictors using multinomial logistic regression. Our findings demonstrate that salary was a factor with statistical significance (*P*< 0.01).

#### Intention to leave in the future

If given the opportunity, 66% of respondents indicated that they would leave their current job for a position in a different district, 67% would leave their current job to take up a job outside of a health facility, and 72% would take up a job outside of Kenya (Table 5). In this case, outside the health facility means working within or outside the health sector but not as a health service provider, for example, not as a nurse providing bed-side nursing. Intention to work in a different district was higher in Turkana (88%) than in Machakos (62%) and Nairobi (57%) (*P*< 0.0001). Turkana also had the highest proportion of health workers who would take a job outside of a health facility (82%), in comparison to Machakos (66%) and Nairobi (59%) (*P* < 0.001). There were no significant differences across the three sites in HCWs’ desire to accept jobs outside of Kenya.

**Table 5:** Percentage distribution of factors related to intent to leave by region

		**Nairobi (n = 164) %**	**Machakos (n = 130) %**	**Turkana (n = 95) %**	**Total (n = 389) %**
Given the opportunity, would leave current job to take a job in a different district	Yes	56.7	62.3	88.4	66.3
	No	43.3	37.7	11.6	33.7
Given the opportunity, would leave current job to take a job outside of a health facility	Yes	58.8	66.4	81.9	66.9
	No	41.2	33.6	18.1	33.1
Given the opportunity, would take a job outside of Kenya	Yes	73	72	71.6	72.3
	No	27	28	28.4	27.7

#### Work preference for different types of health facilities

If given the opportunity to choose, 50.9% of the respondents said they would prefer to work for an NGO, 26.9% would prefer Government, 11.7% out of the country, 6.3% would choose a faith-based organization (FBO) and 4.2% would work for a private institution (data not shown). The preference to work for an NGO was higher in Nairobi, compared to Machakos and Turkana. The preference to work for Government was more common in Turkana, compared to Machakos and Nairobi.

### Comparisons by type of health facility

#### Job satisfaction

A higher proportion of respondents from Government facilities agreed or strongly agreed that they knew what was expected of them at work compared to those in private facilities (85.8% versus 79.7%, *P* = 0.009; Table 6). Private institution employees were more likely to enjoy their work than those in Government (58.2% versus 51.3%, *P* = 0.0004).

**Table 6:** Satisfaction factors that differed between government and private/nongovernmental facilities

		**Government (n = 189) %**	**Private/NGO (n = 211) %**
When I come to work, I know what is expected of me	Strongly disagree	0.5	0.0
	Disagree	1.1	2.4
	Neutral	12.7	17.5
	Agree	42.9	25.9
	Strongly agree	42.9	53.8
I find my work at this facility to be enjoyable	Strongly disagree	2.6	5.2
	Disagree	15.9	8.1
	Neutral	30.2	28.4
	Agree	34.9	33.6
	Strongly agree	16.4	24.6
		(n = 166)	(n = 180)
		%	%
I would encourage my friends and family to seek care here	Strongly disagree	4.2	2.8
	Disagree	6.6	3.9
	Neutral	12.7	13.3
	Agree	42.8	31.1
	Strongly agree	33.7	48.9

Respondents working in private health facilities indicated that they agree or strongly agree to encourage family and friends to seek care at their place of work compared (80% versus 76.5%, *P* = 0.0045; Table 6).

#### Work environment

**Figure 3:**
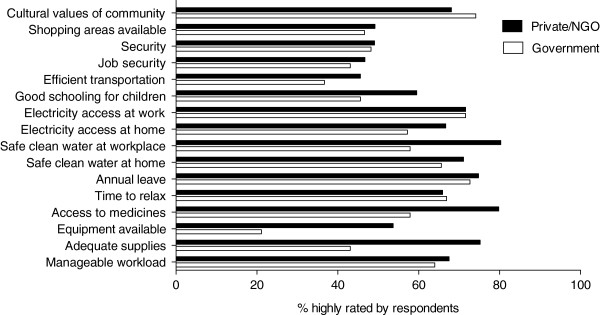
**Comparison of work environment factors between private/nongovernmental organization versus government health facilities.** Percent of healthcare workers in private/NGO and government health facilities who rated various work environment factors favorably.

Results indicate that in almost all aspects, respondents from private institutions rated their environment more favorably than those in Government (Figure 3). Among the most striking differences were adequacy of supplies (76.2% versus 43.1%, *P*< 0.001), good access to drugs (79.8% versus 57.9%, *P*< 0.0001) and safe and clean water at work places (80.3% versus 57.9%), at private and NGO versus public facilities, respectively.

#### Remuneration and compensation factors

Government and private health facilities differed with respect to all aspects of remuneration and compensation that were evaluated. These include reporting of fair salary (10.6% versus 32.1%, *P*< 0.0001), promotion opportunities (36% versus 34%, *P* = 0.019) and stagnation (41.7% versus 24.9%, *P* = 0.022) in government and private/NGO facilities, respectively [Additional file 3].

### Insights from qualitative interviews

Analysis of the results from the qualitative interviews elaborated in matrix form (Table 7) reveals the following themes and typologies:

**Table 7:** Summary of thematic issues emanating from focus group discussions (FDGs) in the three regions

	**Kibera**	**Machakos**	**Turkana**
1. Positive issues about the current work environment	General satisfaction with salary, and job security	Positive response from patients	Good connections, relations with the community
2. Limitations	Discrimination in training, Poor communication from superiors on job assignments; Tribalism.	Low/substandard housing, quality of accommodation.	Limited choices for education facilities for children of staff; language barrier especially for non-locals, unreliable transport to work and lack of electricity.
3. Reasons for leaving job	Stagnation on current job, rivalry between different job cadres especially between Clinical Officers and Nurses	Poor commuter allowance, Huge workload; Government bureaucracy (lack of commodities and other supplies)	Harsh geographical and climatic conditions
4. Retention: What would keep you in your job?	Regular training; good supervision	Better salaries, on-the-job training; more staff to support high workload	Hardship allowances; better accommodation and infrastructure.
5. Compensation factors	Increase allowances (medical, house, overtime and leave allowances)	Need to increase salaries and to pay salaries on time;	Lack of National Social Security Fund (NSSF) and retirement benefits (For private facilities);
6. Gender issues at work	Increase duration of maternity leave	Not very vocal on gender issues, though a few muted voices of females felt that need to increase duration of maternity leave	Men require paternity leave
7. Cultural issues	Men control family planning and especially reproductive health issues sometimes to the detriment of women	A significant number of people believe in witchcraft and use herbs and charms to treat diseases	Women do not easily allow male nurses to attend to them; Women are encouraged to give birth in standing position; New mothers do not breastfeed for a while if they give birth at night.
8. Organizational support	Multiple reporting lines and supervision make it difficult to coordinate work	In-charges are often significantly older than those they supervise and do not fully appreciate their younger colleagues.	Staff are committed to work in hardship conditions

• Several partners are involved in hiring of HCWs and these differ by region.

• For Turkana, these partner organizations comprise more of the faith-based and NGOs. Some of these are AMREF, Merlin, Africa Inland Church (AIC), International Rescue Committee (IRC). Nevertheless, there is also a significant presence of, and Government of Kenya health facilities including the Economic Stimulus Package (ESP) programme and the United States Agency for International Development (USAID) - funded Capacity Kenya project. For Kibera in Nairobi, the organizations managing the facilities comprise mainly the Nairobi City Council (NCC), private providers, and a number of NGOs such as AMREF and *MédecinsSansFrontières* (MSF). For Machakos region, most of the health facilities visited are under the management of the Government

• Getting a job in Turkana is a strategy for gaining Government of Kenya employment with an intention to move out to other regions later or join the health NGOs and or faith-based organizations operating within Turkana

• Inadequate staff, transport, inadequate supportive supervision, essentials (gloves) contribute to dissatisfaction by health care workers (HCWs)

• Lack of housing, payment of support staff, physical state of health facilities contribute to non-conducive environment for HCWs

• Allowances (hardship, marriage, overtime), rest and recuperation are important

• Gender balance in nursing staff (especially in Turkana), as well as cultural issues are critical.

More importantly for health care workers in the Kibera informal settlement, the qualitative interviews reveal a general satisfaction with salary, possibly because the majority are employed in the private sector and NGO facilities where salaries are relatively higher. Beyond salary, what also emerges from the results of the qualitative interviews is that health care workers in the informal settlement are more concerned with management and motivational issues - allowances, mentoring support from the supervisor, and stagnation with regard to promotion.

### Causal analysis

#### Factors affecting health worker motivation

Using bivariate logistic regression, workload with an odds ratio of 5.1 (CI = 2.1 to 12.0) and salary with an odds ratio of 13.5 (CI = 4.315 to 42.185) were the two statistically significant factors (at the 5% level) that were found to affect job satisfaction. The confidence intervals were, however, too wide to consider salary in particular as an important predictor of motivation in this case. Hence, a larger sample size may be required in subsequent studies.

## Discussion

Findings from this study confirm distinct issues relating to motivation and retention in each of the three settings and can inform evidence-based policy-making and programming as devolved County Governments strengthen their health workforce. Differences between private and public health facilities are also illustrated in this study and should also be taken into consideration. The key issues which have emerged as bearing policy implications are discussed below.

### Gender imbalance in the Turkana health workforce

Level of education and equality in gender distribution were significantly lower in Turkana than in Kibera or Machakos. It is estimated that on the national level, females make up about 53% of the health workforce [24]. The fact that female HCWs in Turkana only make up 30% of the workforce may be attributable to the difficult environmental conditions within the region deemed unfavorable to women HCWs. This gender imbalance has serious cultural implications for service delivery. A large barrier to skilled delivery in health facilities is the unwillingness of women to be examined and have their child delivered by a male service provider [25]. With 70% of health workers being male, cultural inhibitions are bound to continue and present a barrier to health equity [26]. The observed gender imbalance is not currently addressed by Kenya’s HRH policy and should inform county policy on recruitment, particularly in the hard-to-reach areas. In-depth yet rapid reviews of gender dynamics in primary health care facilities in these regions, Turkana County in particular, followed by development and implementation of gender mainstreaming strategies should be undertaken to address the issue.

### Education and training gaps

A much smaller proportion of HCWs in Turkana reported feeling adequately trained for their jobs than in the other two regions. This correlates with our findings that low levels of education and opportunities for professional upgrading courses were reported in Turkana. During a study on the health workforce in Uganda, HCWs disclosed that training provided significant reward and motivation [15,19]. Inadequate skills among health workers, therefore, not only affect quality of services provided, but have direct implications on the motivation and retention of health workers. A comprehensive and equitable continuous training programme for health workers is important. We recommend that tailored training packages and strategies be developed and implemented in hard-to-reach areas, such as Turkana, in order to address the training gaps identified in this study.

### A need for improved job satisfaction

The findings from this study show that levels of satisfaction differ on some attributes between the three regions on the one hand, and on the other between public and private health facilities. Staff shortages, transportation, inadequate supportive supervision, and a lack of essential supplies and functional equipment are notable factors of the working environment that contribute to dissatisfaction. Allowances (hardship, marriage, over-time), rest and recuperation are also important compensation aspects that would affect satisfaction. This study identified adequate training, job security, salary, and supervisory support as critical satisfaction factors, as others have also identified in other settings [8,19,23]. Additionally, the finding in this study that having a manageable workload was a significant contributor to job satisfaction, which is related to motivation, supports existing literature [27]. A high proportion of health workers in Turkana feel that their workload is not manageable. This could be related to greater staff shortages in Turkana, as observed in our findings. Health workers in these settings also reported to taking on additional duties due to inadequate human resource capacity [28]. This presents an additional problem of workers completing tasks for which they lack skills and are not trained.

### Work environment, remuneration, compensation and retention

Inadequate access to electricity, equipment and transportation was found to be most critical in Turkana, as expected. Lack of housing, inadequate payment of support staff, and poor physical state of the health facility contribute to a non-conducive working environment. More than 30% of all health workers surveyed did not feel that they had job security. The working environment in private facilities was rated higher than that in Government facilities. Inadequate working conditions, coupled with low job satisfaction and stability, are bound to demotivate health workers and impact retention [4,5].

Remuneration is a critical factor of motivation and retention. Yet, a high proportion of health staff, particularly in Machakos, feel that their remuneration is not fair. Machakos was also found to have the highest rate of attrition. Opportunities for promotion or career growth are key elements of motivation [5]. Family health care, salary, and terminal benefits are important compensation factors that are closely linked to motivation and retention. Health workers place emphasis on family care; compensation is highly regarded if it has direct benefit to dependents. Family health care is rated higher than salary among respondents. The majority of health workers in this study were in their mid-years and married with children. Compensation to workers with families transcends individual interests and, therefore, policies on recruitment and compensation should include benefits for dependents.

Overall, 13% of respondents had changed jobs in the last 12 months before the survey and 20% indicated that they intend to leave their current job within two years. Attrition rates are highest in Machakos and Nairobi compared to Turkana. Although fewer workers had reported to having changed jobs in the past year, this may be due to low opportunities for alternative employment in this region. Further, a higher proportion of health workers in Turkana would leave their job for another district signifying lower levels of satisfaction, likely due to the relatively poorer working conditions in this region. Additionally, qualitative interviews revealed that HCWs in Turkana seek employment with the Government intending to relocate to other regions after being hired. This is similar to a study on motivation of health workers in Uganda in which 20% indicated that they could leave within three years [23]. In Uganda, however, the average number of years spent in the job was much higher than that observed in this study. In Uganda, the average years of stay were ten compared to five years in Kenya. This was attributed to the high status accorded to health sector jobs, as well as stable and reasonable compensation. Our study findings also indicate that salary is an important predictor of health worker retention. Health workers are likely to move from Government health facilities to those operated by NGOs, private facilities and out of the country in search for a better working environment.

Although not highlighted in this study, variations in what are considered the most important motivational factors between different types of health care professionals needs to be considered. A study to determine policies to improve nurse recruitment and retention in rural Kenya has identified a number of job attributes that can be directly influenced by health policy in order to increase attraction to rural postings. These include permanent contracts linked to rural posts, allowances, opportunities for training, and reduced years of experience before being promoted [7]; more recent studies [29,30] also suggest additional strategies. These results show that nurses place the highest value on attributes that would be expected to have immediate monetary advantages such as salary enhancement or long-term factors (promotion, training and permanent contract). A study conducted in rural Ghana investigated the factors related to low retention of health workers [31]. For doctors, although salary is important, it is more the career development concerns which keep them in urban areas. The study also shows that short-term service in rural areas would be preferable if it was linked to coaching and mentoring, as well as to career growth.

### Recommendations

The findings of this study and the policy implications enumerated above enable the delineation of several recommendations. First, retention schemes that are tailored to each of the three regions need to be developed and applied through decentralized HRH management systems within the regions that can address their unique issues. Secondly, competitive compensation packages should be developed for health staff, particularly in hard-to-reach areas. These packages should include family health care and be reviewed regularly in order to address rapidly changing needs and circumstances over time. Thirdly, strategies for career growth and promotion, especially for the higher cadre of health workers, such as doctors should be developed. Fourth, the establishment of a model ‘HRH community’ by developing and marketing a funding proposal to establish such a model within selected NGO/FBO health facilities in hard-to-reach areas would be beneficial. The case for the model HRH community is based on one of the important insights learnt from these findings: that there is need to demonstrate that addressing HRH issues such as those related to the work environment and employee satisfaction works, and that indeed there are solutions. Infrastructure and motivational as well as job satisfaction factors that cater for the needs of not just health workers but those of their families and dependants are important. Unlike the current practice where the focus is on the increase in the number of service providers per population, there is need to link increased investment in HRH to increased productivity and performance. As more health service providers are recruited for each health facility, we should be able to see a corresponding improvement in health indicators in the catchment of the facility. Fifth, there is a need to conduct a more focused and detailed follow-up retrospective longitudinal study to determine the factors related to career transitions among primary-level service providers in the three regions. Lastly, many factors have been identified by health workers as motivational or important for their job satisfaction. This makes it difficult from a policy perspective to choose the appropriate combination of incentives, benefits, and other interventions to implement. It is therefore recommended that a discrete choice experiment be conducted.

### Limitations of the study

One of the limitations of the study is that it employed a cluster sample design which involves a higher degree of sample error. Nevertheless, this disadvantage was offset by including the design effect into the calculation of the sample size. Secondly, in this study, a high proportion of health care workers interviewed were support staff who cannot really be categorized as technical service providers. Nevertheless, this is the reality and universe of the staff complement working in the primary health care facilities surveyed. Thirdly, even the professional groups of health care workers are not homogeneous but cover different varieties of specialists (nurses, laboratory technicians and technologists, pharmacists, and so on) who strictly speaking should not always be consolidated into one group during analysis. A follow-up survey has been completed (December 2014 to January 2014) and the report prepared (April 2014) awaits further analysis. Further analysis of data in this follow-up survey, in which support staff represent a minimal proportion of respondents (3%), is expected to generate richer evidence due to the larger sample size for each professional cadre of health care workers.

## Conclusions

The findings from this study indeed confirm distinct issues related to motivation and retention in each of the three settings that the study was conducted in. Secondly, motivation and retention in the three regions is associated with particular background characteristics of health workers. Thirdly, results of this study show that salary is an important predictor of motivation and retention of health workers. Lastly, insights from this study show that there are solutions to HRH issues such as those related to employee satisfaction and work environment. The findings and recommendations from this study can directly inform and influence the review of the Kenya HRH Strategic Plan and be employed to strengthen HRH systems in County Governments as devolution takes shape.

## Competing interests

The authors declare that they have no competing interests.

## Authors’ contributions

(DO) was the principal investigator who steered the study from the development of the proposal through to project start-up, implementation, data collection and analysis, and manuscript development. (SO) is the project manager and supported the development of tables and figures, discussion and conclusion, as well as the manuscript review. (JJ) developed and wrote this manuscript using data and descriptions from the main report for this project. All authors read and approved the final manuscript.

## Supplementary Material

Additional file 1 Percentage distribution of job satisfaction factors by region. Click here for file

Additional file 2 Distribution of factors related to the work environment by region. Click here for file

Additional file 3 Comparison of compensation factors by type of facility. Click here for file
