# Supplementary material for: Factors affecting motivation and retention of primary health care workers in three disparate regions in Kenya
Source: Hum Resour Health. 2014 Jun 6;12:33. doi: 10.1186/1478-4491-12-33 (PMC4097093; doi:10.1186/1478-4491-12-33)
Supplement: Additional file 1 — Percentage distribution of job satisfaction factors by region. [file 1478-4491-12-33-S1.pdf]

ADDITIONAL FILES

Additional File 1: Percentage Distribution of Job Satisfaction Factors by Region

		County								
		Nairobi		Machakos		Turkana		Total		
		n	%	n	%	n	%	n	%	p-value
Considering everything, I am satisfied with my job.	Strongly disagree	10	6.0	13	9.6	2	2.0	25	6.2	0.163
	Disagree	31	18.5	29	21.5	23	23.2	83	20.6	
	Neutral	49	29.2	44	32.6	23	23.2	116	28.9	
	Agree	44	26.2	31	23.0	31	31.3	106	26.4	
	Strongly Agree	34	20.2	18	13.3	20	20.2	72	17.9	
The job is a good match for my skills and experience.	Strongly disagree	5	3.0	9	6.8	1	1.0	15	3.8	0.028
	Disagree	20	11.9	25	18.8	7	7.1	52	13.0	
	Neutral	33	19.6	18	13.5	23	23.5	74	18.5	
	Agree	65	38.7	53	39.8	46	46.9	164	41.1	
	Strongly Agree	45	26.8	28	21.1	21	21.4	94	23.6	
When I come to work, I know what is expected of me.	Strongly disagree	1	0.6	0	0	0	0	1	0.2	0.002
	Disagree	1	0.6	4	3.0	3	3.0	8	2.0	
	Neutral	22	13.0	22	16.4	17	17.2	61	15.2	
	Agree	39	23.1	54	40.3	43	43.4	136	33.8	
	Strongly Agree	105	62.1	54	40.3	36	36.4	195	48.5	
I receive recognition for doing good work.	Strongly disagree	17	10.1	11	8.1	4	4.0	32	8.0	0.001
	Disagree	17	10.1	30	22.2	6	6.1	53	13.2	
	Neutral	40	23.8	40	29.6	30	30.3	110	27.4	
	Agree	51	30.4	33	24.4	39	39.4	123	30.6	
	Strongly Agree	43	25.6	21	15.6	20	20.2	84	20.9	
My immediate supervisor cares about me as a person	Strongly disagree	4	2.6	3	2.7	2	2.5	9	2.6	0.183
	Disagree	10	6.6	4	3.5	1	1.3	15	4.3	
	Neutral	26	17.1	20	17.7	18	22.5	64	18.6	
	Agree	49	32.2	52	46.0	35	43.8	136	39.4	
	Strongly Agree	63	41.4	34	30.1	24	30.0	121	35.1	

		County								
		Nairobi		Machakos		Turkana		Total		
		n	%	n	%	n	%	n	%	p-value
My supervisor provides me with support and encouragement.	Strongly disagree	5	3.0	5	3.7	3	3.0	13	3.2	0.430
	Disagree	14	8.3	12	8.9	6	6.1	32	7.9	
	Neutral	34	20.1	32	23.7	28	28.3	94	23.3	
	Agree	57	33.7	54	40.0	39	39.4	150	37.2	
	Strongly Agree	59	34.9	32	23.7	23	23.2	114	28.3	
Someone talks to me regularly to encourage my development	Strongly disagree	7	4.1	10	7.4	3	3.1	20	5.0	0.009
	Disagree	33	19.5	23	17.0	3	3.1	59	14.7	
	Neutral	49	29.0	34	25.2	38	38.8	121	30.1	
	Agree	49	29.0	46	34.1	38	38.8	133	33.1	
	Strongly Agree	31	18.3	22	16.3	16	16.3	69	17.2	
Overall, the morale level at my department or section is good.	Strongly disagree	7	4.2	8	5.9	4	4.0	19	4.7	0.600
	Disagree	19	11.3	25	18.5	19	19.2	63	15.7	
	Neutral	48	28.6	36	26.7	28	28.3	112	27.9	
	Agree	65	38.7	49	36.3	31	31.3	145	36.1	
	Strongly Agree	29	17.3	17	12.6	17	17.2	63	15.7	
My opinion seems to matter at work; I am respected.	Strongly disagree	2	1.2	4	3.0	4	4.1	10	2.5	0.213
	Disagree	16	9.7	19	14.1	3	3.1	38	9.5	
	Neutral	46	27.9	33	24.4	28	28.6	107	26.9	
	Agree	72	43.6	52	38.5	44	44.9	168	42.2	
	Strongly Agree	29	17.6	27	20.0	19	19.4	75	18.8	
I have at least one good friend at work.	Strongly disagree	1	0.6	4	3.0	1	1.0	6	1.5	0.030
	Disagree	10	6.0	7	5.2	1	1.0	18	4.5	
	Neutral	21	12.7	27	20.0	20	20.2	68	17.0	
	Agree	65	39.2	52	38.5	51	51.5	168	42.0	
	Strongly Agree	69	41.6	45	33.3	26	26.3	140	35.0	

		County								
		Nairobi		Machakos		Turkana		Total		
		n	%	n	%	n	%	n	%	p-value
I would encourage my friends and family to seek care here.	Strongly disagree	3	2.0	7	6.2	2	2.5	12	3.5	0.023
	Disagree	6	3.9	8	7.1	4	4.9	18	5.2	
	Neutral	26	17.1	13	11.5	6	7.4	45	13.0	
	Agree	45	29.6	41	36.3	41	50.6	127	36.7	
	Strongly Agree	72	47.4	44	38.9	28	34.6	144	41.6	
I have flexibility to balance the demands of my workplace and my personal life.	Strongly disagree	2	1.2	9	6.7	3	3.0	14	3.5	0.137
	Disagree	29	17.4	22	16.3	11	11.1	62	15.5	
	Neutral	41	24.6	37	27.4	27	27.3	105	26.2	
	Agree	66	39.5	48	35.6	34	34.3	148	36.9	
	Strongly Agree	29	17.4	19	14.1	24	24.2	72	18.0	
I find my work at this facility to be enjoyable.	Strongly disagree	7	4.2	6	4.5	4	4.0	17	4.2	0.628
	Disagree	18	10.7	17	12.7	12	12.1	47	11.7	
	Neutral	40	23.8	43	32.1	34	34.3	117	29.2	
	Agree	67	39.9	40	29.9	30	30.3	137	34.2	
	Strongly Agree	36	21.4	28	20.9	19	19.2	83	20.7	
My supervisors are kind to me; they do not verbally, physically or emotionally abuse	Strongly disagree	3	1.8	5	3.7	1	1.0	9	2.2	0.013
	Disagree	7	4.2	9	6.7	3	3.0	19	4.7	
	Neutral	30	17.9	31	23.0	35	35.4	96	23.9	
	Agree	60	35.7	45	33.3	40	40.4	145	36.1	
	Strongly Agree	68	40.5	44	32.6	20	20.2	132	32.8	
	31	0	0	1	0.7	0	0	1	0.2	
My peers are kind to me; they do not verbally, physically or emotionally abuse me.	Strongly disagree	3	1.8	2	1.5	0	0	5	1.2	0.232
	Disagree	11	6.5	10	7.4	3	3.0	24	6.0	
	Neutral	32	18.9	28	20.7	29	29.3	89	22.1	
	Agree	60	35.5	49	36.3	42	42.4	151	37.5	
	Strongly Agree	63	37.3	46	34.1	25	25.3	134	33.3	
My patients and their family	Strongly disagree	6	3.6	2	1.5	2	2.0	10	2.5	0.584

		County								
		Nairobi		Machakos		Turkana		Total		
		n	%	n	%	n	%	n	%	p-value
members are kind to me; they do not verbally, physically or	Disagree	18	10.7	10	7.4	4	4.0	32	8.0	
	Neutral	34	20.2	30	22.2	27	27.3	91	22.6	
	Agree	62	36.9	54	40.0	38	38.4	154	38.3	
	Strongly Agree	48	28.6	39	28.9	28	28.3	115	28.6	
I have been provided the training needed to succeed in my position.(on the job	Strongly disagree	7	4.2	14	10.4	15	15.2	36	9.0	<0.0001
	Disagree	27	16.1	29	21.5	25	25.3	81	20.1	
	Neutral	32	19.0	33	24.4	32	32.3	97	24.1	
	Agree	52	31.0	37	27.4	10	10.1	99	24.6	
	Strongly Agree	49	29.2	22	16.3	17	17.2	88	21.9	
	33	1	0.6	0	0	0	0	1	0.2	
The organization takes specific measures to protect me against HIV/AIDS and TB.	Strongly disagree	6	3.6	12	9.0	7	7.1	25	6.2	0.313
	Disagree	15	8.9	14	10.4	8	8.1	37	9.2	
	Neutral	37	21.9	36	26.9	27	27.3	100	24.9	
	Agree	58	34.3	45	33.6	35	35.4	138	34.3	
	Strongly Agree	53	31.4	27	20.1	22	22.2	102	25.4	
I consider myself a part of this community.	Strongly disagree	0	0	1	0.7	0	0	1	0.2	0.805
	Disagree	7	4.2	8	5.9	4	4.0	19	4.7	
	Neutral	35	20.8	30	22.2	20	20.2	85	21.1	
	Agree	68	40.5	46	34.1	34	34.3	148	36.8	
	Strongly Agree	57	33.9	50	37.0	41	41.4	148	36.8	
	32	1	0.6	0	0	0	0	1	0.2	
I am fairly evaluated on my work.	Strongly disagree	0	0	7	5.3	7	7.1	14	3.5	0.002
	Disagree	10	6.0	16	12.1	2	2.0	28	7.1	
	Neutral	39	23.5	33	25.0	27	27.3	99	24.9	
	Agree	70	42.2	51	38.6	45	45.5	166	41.8	
	Strongly Agree	47	28.3	25	18.9	18	18.2	90	22.7	
The in charge here is competent and	Strongly disagree	1	0.7	6	5.3	3	3.7	10	2.9	0.060

		County								
		Nairobi		Machakos		Turkana		Total		
		n	%	n	%	n	%	n	%	p-value
	Neutral	13	8.6	9	8.0	12	14.8	34	9.9	
	Agree	61	40.4	38	33.6	38	46.9	137	39.7	
	Strongly Agree	69	45.7	53	46.9	27	33.3	149	43.2	
I am actively involved in helping to make this a great health care facility.	Strongly disagree	1	0.7	2	1.8	1	1.2	4	1.2	0.426
	Disagree	3	2.0	6	5.3	0	0	9	2.6	
	Neutral	13	8.5	7	6.2	7	8.6	27	7.8	
	Agree	60	39.2	50	44.2	36	44.4	146	42.1	
	Strongly Agree	76	49.7	48	42.5	37	45.7	161	46.4	
