# Supplementary material for: Factors affecting motivation and retention of primary health care workers in three disparate regions in Kenya
Source: Hum Resour Health. 2014 Jun 6;12:33. doi: 10.1186/1478-4491-12-33 (PMC4097093; doi:10.1186/1478-4491-12-33)
Supplement: Additional file 2 — Distribution of factors related to the work environment by region. [file 1478-4491-12-33-S2.pdf]

Additional File 2: Distribution of Factors Related to the Work Environment by Region

		County								
		Nairobi		Machakos		Turkana		Total		
		n	%	n	%	n	%	n	%	p-value
The workload is manageable.	Strongly disagree	4	2.4	16	12.0	5	5.1	25	6.2	0.001
	Disagree	21	12.4	19	14.3	21	21.2	61	15.2	
	Neutral	25	14.7	22	16.5	4	4.0	51	12.7	
	Agree	92	54.1	57	42.9	47	47.5	196	48.8	
	Strongly Agree	28	16.5	19	14.3	22	22.2	69	17.2	
Adequate supplies (gloves, needles, bandages, etc).	Strongly disagree	6	3.5	17	12.6	12	12.1	35	8.6	<0.0001
	Disagree	18	10.5	33	24.4	16	16.2	67	16.5	
	Neutral	26	15.2	23	17.0	10	10.1	59	14.6	
	Agree	53	31.0	36	26.7	29	29.3	118	29.1	
	Strongly Agree	68	39.8	26	19.3	32	32.3	126	31.1	
Equipment available	Strongly disagree	18	11.3	28	21.1	32	32.7	78	19.9	<0.0001
	Disagree	19	11.9	42	31.6	30	30.6	91	23.3	
	Neutral	39	24.4	23	17.3	11	11.2	73	18.7	
	Agree	52	32.5	33	24.8	15	15.3	100	25.6	
	Strongly Agree	32	20.0	7	5.3	10	10.2	49	12.5	
Good access to drugs and medications.	Strongly disagree	2	1.3	6	5.3	3	3.7	11	3.2	<0.0001
	Disagree	10	6.5	27	23.9	7	8.6	44	12.6	
	Neutral	20	12.9	25	22.1	7	8.6	52	14.9	
	Agree	53	34.2	34	30.1	40	49.4	127	36.4	
	Strongly Agree	70	45.2	21	18.6	24	29.6	115	33.0	
My job allows me to take time to relax during the lunch break.	Strongly disagree	4	2.6	13	11.5	2	2.5	19	5.5	0.002
	Disagree	21	13.6	24	21.2	10	12.3	55	15.8	
	Neutral	27	17.5	10	8.8	6	7.4	43	12.4	
	Agree	55	35.7	41	36.3	38	46.9	134	38.5	
	Strongly Agree	47	30.5	25	22.1	25	30.9	97	27.9	

		County								
		Nairobi		Machakos		Turkana		Total		
		n	%	n	%	n	%	n	%	p-value
I am allowed to go on leave	Strongly disagree	5	3.2	16	14.2	6	7.5	27	7.8	<0.0001
	Disagree	8	5.2	14	12.4	14	17.5	36	10.3	
	Neutral	12	7.7	12	10.6	4	5.0	28	8.0	
	Agree	40	25.8	31	27.4	29	36.3	100	28.7	
	Strongly Agree	90	58.1	40	35.4	27	33.8	157	45.1	
At my residence, I have access to safe, clean water.	Strongly disagree	4	2.3	16	11.9	12	12.2	32	7.9	<0.0001
	Disagree	17	9.9	28	20.7	13	13.3	58	14.4	
	Neutral	16	9.4	10	7.4	11	11.2	37	9.2	
	Agree	56	32.7	45	33.3	36	36.7	137	33.9	
	Strongly Agree	78	45.6	35	25.9	26	26.5	139	34.4	
	34	0	0	1	0.7	0	0	1	0.2	
At work, I have access to safe, clean water.	Strongly disagree	7	4.1	14	10.4	10	10.1	31	7.7	<0.0001
	Disagree	8	4.7	36	26.7	12	12.1	56	13.8	
	Neutral	15	8.8	11	8.1	10	10.1	36	8.9	
	Agree	57	33.3	43	31.9	37	37.4	137	33.8	
	Strongly Agree	84	49.1	31	23.0	30	30.3	145	35.8	
At residence, I have good access to electricity	Strongly disagree	3	1.9	19	16.8	39	48.1	61	17.5	<0.0001
	Disagree	8	5.2	16	14.2	30	37.0	54	15.5	
	Neutral	10	6.5	5	4.4	2	2.5	17	4.9	
	Agree	54	34.8	33	29.2	9	11.1	96	27.5	
	Strongly Agree	80	51.6	40	35.4	1	1.2	121	34.7	
At work, I have good access to electricity.	Strongly disagree	4	2.4	10	7.4	32	32.3	46	11.4	<0.0001
	Disagree	6	3.6	7	5.2	22	22.2	35	8.7	
	Neutral	18	10.7	9	6.7	7	7.1	34	8.5	
	Agree	55	32.7	40	29.6	23	23.2	118	29.4	
	Strongly Agree	85	50.6	69	51.1	15	15.2	169	42.0	
I have access to good schooling for my children.	Strongly disagree	11	7.3	23	18.3	18	18.6	52	13.9	<0.0001
	Disagree	11	7.3	26	20.6	11	11.3	48	12.8	
	Neutral	34	22.5	29	23.0	14	14.4	77	20.6	
	Agree	57	37.7	34	27.0	33	34.0	124	33.2	
	Strongly Agree	38	25.2	14	11.1	21	21.6	73	19.5	

		County								
		Nairobi		Machakos		Turkana		Total		
		n	%	n	%	n	%	n	%	p-value
I have safe and efficient transportation to work	Strongly disagree	18	11.0	22	16.3	23	23.7	63	15.9	<0.0001
	Disagree	21	12.9	38	28.1	35	36.1	94	23.8	
	Neutral	42	25.8	21	15.6	12	12.4	75	19.0	
	Agree	54	33.1	35	25.9	14	14.4	103	26.1	
	Strongly Agree	28	17.2	19	14.1	13	13.4	60	15.2	
I feel I have job security	Strongly disagree	23	13.8	27	20.0	18	18.2	68	17.0	0.042
	Disagree	23	13.8	34	25.2	20	20.2	77	19.2	
	Neutral	35	21.0	25	18.5	16	16.2	76	19.0	
	Agree	61	36.5	26	19.3	27	27.3	114	28.4	
	Strongly Agree	25	15.0	23	17.0	18	18.2	66	16.5	
I feel there is adequate security at my residence and within the surrounding	Strongly disagree	14	9.2	14	12.4	12	14.8	40	11.5	0.004
	Disagree	24	15.7	31	27.4	14	17.3	69	19.9	
	Neutral	45	29.4	12	10.6	12	14.8	69	19.9	
	Agree	46	30.1	40	35.4	24	29.6	110	31.7	
	Strongly Agree	24	15.7	16	14.2	19	23.5	59	17.0	
The community where I live has good shopping areas and entertainment.	Strongly disagree	10	6.6	17	15.0	12	15.0	39	11.3	0.008
	Disagree	23	15.2	24	21.2	19	23.8	66	19.2	
	Neutral	46	30.5	13	11.5	15	18.8	74	21.5	
	Agree	47	31.1	43	38.1	24	30.0	114	33.1	
	Strongly Agree	25	16.6	16	14.2	10	12.5	51	14.8	
I understand and appreciate the cultural values within the surrounding community	Strongly disagree	5	3.0	6	4.5	1	1.0	12	3.0	<0.0001
	Disagree	14	8.3	15	11.2	1	1.0	30	7.5	
	Neutral	42	25.0	21	15.7	11	11.2	74	18.5	
	Agree	69	41.1	64	47.8	41	41.8	174	43.5	
	Strongly Agree	38	22.6	28	20.9	44	44.9	110	27.5	
