# Supplementary material for: Factors affecting motivation and retention of primary health care workers in three disparate regions in Kenya
Source: Hum Resour Health. 2014 Jun 6;12:33. doi: 10.1186/1478-4491-12-33 (PMC4097093; doi:10.1186/1478-4491-12-33)
Supplement: Additional file 3 — Comparison of compensation factors by type of facility. [file 1478-4491-12-33-S3.pdf]

Additional File 3: Comparison of Compensation Factors by Type of Facility

		Government		Private/NGO		
		n	%	n	%	p-value
My salary package is fair	Strongly disagree	67	35.4	49	23.1	<0.0001
	Disagree	68	36.0	48	22.6	
	Neutral	34	18.0	47	22.2	
	Agree	14	7.4	54	25.5	
	Strongly Agree	6	3.2	14	6.6	
I feel there are sufficient opportunities for promotion with this employer	Strongly disagree	34	18.0	32	15.1	0.019
	Disagree	51	27.0	39	18.4	
	Neutral	36	19.0	68	32.1	
	Agree	51	27.0	48	22.6	
	Strongly Agree	17	9.0	25	11.8	
I feel there is no stagnation in the Ministry/organization	Strongly disagree	32	19.6	23	12.7	0.022
	Disagree	36	22.1	22	12.2	
	Neutral	39	23.9	56	30.9	
	Agree	37	22.7	56	30.9	
	Strongly Agree	19	11.7	24	13.3	
